# COVID-19 Emergency: Social Distancing and Social Exclusion as Risks for Suicide Ideation and Attempts in Adolescents

**DOI:** 10.3389/fpsyg.2020.551113

**Published:** 2020-11-19

**Authors:** Claudio Longobardi, Rosalba Morese, Matteo Angelo Fabris

**Affiliations:** ^1^Department of Psychology, University of Turin, Turin, Italy; ^2^Faculty of Communication, Cultural and Society, Università della Svizzera Italiana, Lugano, Switzerland; ^3^Faculty of Biomedical Sciences, Institute of Public Health, Università della Svizzera Italiana, Lugano, Switzerland

**Keywords:** coronavirus emergency, social exclusion, social distancing, suicide ideation, adolescents, suicide attempts, COVID-19

The world is in the middle of tackling the challenge of the coronavirus emergency. On March 11, 2020, the World Health Organization (WHO) declared a pandemic, and Italy was among the nations most affected, with more than 29,000 victims (European Centre for Disease Prevention and Control, 2020; WHO, 2020). Measures to counter the progression of the epidemic have forced a review and reformulation of the day-to-day activities of the affected populations, necessitating restrictive measures such as social distancing and quarantine.

Several studies have hypothesized that quarantine could have a negative psychological impact on the population (Brooks et al., 2020). Studies have shown that quarantine leads to a decrease in positive emotions and an increase in negative emotions, such as anger and fear (Cava et al., 2005).

The experience of quarantine tends to correlate with decreased psychological well-being and the onset of psychological symptoms and emotional disorders, such as depression, anxiety, insomnia, and post-traumatic symptoms (Brooks et al., 2020). Factors such as the quarantine duration, the uncertainty of information, and the fear of being infected or of the infection of loved ones appear to be factors that increase distress (Robertson et al., 2004; Reynolds et al., 2008; Brooks et al., 2020). In addition, the loss of routine and confinement, which causes a drastic reduction in physical and social contact with others, can increase the sense of isolation and loneliness, resulting in psychological distress (Brooks et al., 2020). The literature has focused mainly on the psychological well-being of adults and health professionals, and not on adolescent well-being, and, in particular, the risk of suicidal ideation.

Suicide is estimated to be the world's second leading cause of death among adolescents, and suicidal ideation, which contributes to the risk of committing suicide, is at its peak in adolescence (Hawton et al., 2012; Uddin et al., 2019).

Adolescence is a delicate period for future psychological adaptation, and it is at this stage of evolution that the need for group membership is strongly expressed (Badenes-Ribera et al., 2019). In agreement with self-determination theory (Deci and Ryan, 1985), adolescents seek to gratify their need to feel socially connected with others in order to satisfy their need to belong to the group, as well as to feel more popular among their peers. The need for belonging is a basic psychological need that if frustrated or not satisfied can lead to a sense of isolation and loneliness that affects adolescent psychological well-being and can help increase the risk of suicide (Stewart et al., 2017). Thwarted belongingness, or the perception that one is alienated from others and not an integral part of a valued social group, is considered a predictive factor for suicidal ideation by suicide theories and, in particular, by the psychological interpersonal theory of suicidal behavior (Joiner et al., 2009). Quarantine seems to have all the characteristics to promote this state of isolation and loneliness. For example, in Italy, schools, as well as facilities dedicated to sports, arts, and leisure activities, have been closed throughout the country since March 10, 2020. In this way, spaces where adolescents tend to congregate have been reduced, thus reducing peer contact. In addition, the closure of schools has resulted in the use of online teaching, which could be a source of stress and disadvantage for some adolescents, and this is interesting in terms of our reflection given that school performance seems to be related to the phenomenon of adolescent suicide (Evans et al., 2004; Stewart et al., 2017). In addition, school closures could result in a lack of access to resources for adolescents with previous psychological difficulties, and this is a critical issue for the mental health of such adolescents.

Of course, young people can use social networks to keep in touch with their peers, but we must warn that the excessive use of social media can increase distress and the risk of victimization and have an impact on the psychological well-being of children (Longobardi et al., 2020a,b). We must also assume that at greater risk will still be the low-rise adolescents in social status such as popularity, rejection, and social exclusion. In fact, the literature seems to indicate that children who are rejected and excluded from their peer groups seem to experience the same dynamic even in the virtual world (Longobardi et al., 2020a). Therefore, the quarantine situation could increase the sense of exclusion and isolation and, in turn, increase suicidal risk (Morese and Longobardi, 2020).

Other factors also need to be taken into account. The quarantine period can help to increase family conflict; there is therefore often increasing distress and a sense of solitude in the families of quarantined children and adolescents. Negative experiences in the family, such as divorce and conflictive parental separation, seem to be vulnerability factors for suicide in adolescence (Hawton et al., 2012). The death of one's relatives is also recognized as a risk factor for suicide in adolescence, and daily reports tell us of mothers, fathers, and grandparents who have died following infection from coronavirus disease 2019 (COVID-19) and family members broken by the event of mourning (Jakobsen and Christiansen, 2011).

In general, this intervention aims to highlight concern for the psychological well-being of adolescents and, in particular, to address the risk of suicidal ideation and behavior. It is important, in our view, to extend knowledge about this phenomenon in relation to quarantine. We also consider it useful to inform psychologists, psychiatrists, social workers, and all the authorities involved in the protection of minors to ensure that they should not forget adolescents and the risk of suicide during this emergency.

It is important to train and inform adolescent psychological well-being facilities, start awareness campaigns, and structure dedicated services that can intercept at-risk cases. In addition, patients already being treated for suicidal behavior should be monitored for ongoing issues, and, if possible, they should conduct their psychological therapies using telematics.

## Author Contributions

CL, RM, and MF conceptualized the contribution. CL wrote the paper. RM reviewed the manuscript. MF provided the critical revision processes as principal investigator (PI). All authors approved the submission of the manuscript.

## Conflict of Interest

The authors declare that the research was conducted in the absence of any commercial or financial relationships that could be construed as a potential conflict of interest.
